# High-Efficiency Visible Transmitting Polarizations Devices Based on the GaN Metasurface

**DOI:** 10.3390/nano8050333

**Published:** 2018-05-15

**Authors:** Zhongyi Guo, Haisheng Xu, Kai Guo, Fei Shen, Hongping Zhou, Qingfeng Zhou, Jun Gao, Zhiping Yin

**Affiliations:** School of Computer and Information, Hefei University of Technology, Hefei 230009, China; xuhaisheng@mail.hfut.edu.cn (H.X.); kai.guo@hfut.edu.cn (K.G.); shenfei@hfut.edu.cn (F.S.); ciangela@hfut.edu.cn (H.Z.); enqfzhou@hfut.edu.cn (Q.Z.); gaojun@hfut.edu.cn (J.G.)

**Keywords:** metasurfaces, orthogonal polarization, high-efficiency, polarization analyzer

## Abstract

Metasurfaces are capable of tailoring the amplitude, phase, and polarization of incident light to design various polarization devices. Here, we propose a metasurface based on the novel dielectric material gallium nitride (GaN) to realize high-efficiency modulation for both of the orthogonal linear polarizations simultaneously in the visible range. Both modulated transmitted phases of the orthogonal linear polarizations can almost span the whole 2π range by tailoring geometric sizes of the GaN nanobricks, while maintaining high values of transmission (almost all over 90%). At the wavelength of 530 nm, we designed and realized the beam splitter and the focusing lenses successfully. To further prove that our proposed method is suitable for arbitrary orthogonal linear polarization, we also designed a three-dimensional (3D) metalens that can simultaneously focus the *X*-, *Y*-, 45°, and 135° linear polarizations on spatially symmetric positions, which can be applied to the linear polarization measurement. Our work provides a possible method to achieve high-efficiency multifunctional optical devices in visible light by extending the modulating dimensions.

## 1. Introduction

Optical metasurfaces, a two-dimensional planar variation of the concept of metamaterials, have been engineered to realize exotic electromagnetic properties by control of the phase, amplitude, and polarization of incident light, which are capable of being manipulated in a desirable manner [1,2]. A metasurface is usually composed of nano-antennas or nano-apertures because of introduced arbitrary abrupt phase shifts by adjusting geometric sizes [3,4,5]. Compared to the conventional optical devices, devices made from metasurfaces have the advantage of ultrathin, highly integrated, versatile, low-cost characteristics [6,7,8]. With the development of nanotechnology, many structures have been realized to demonstrate the broad applications of metasurfaces, including metalens [9,10,11], wave plates [12], filters [13], absorbers [14], vortex beam generation [15], and holograms [16,17].

In the visible light region, much of the previous work was based on metal nanostructures, and most of these had been proved to be low transmission as a result of Joule losses [18,19]. For semiconductor-based dielectric metasurfaces, there is no inherent loss for similar metal nanostructures; therefore these have demonstrated superior performance for providing a high transmission in many applications of metasurface devices to date [20,21,22,23]. At the beginning, a dielectric material was combined with a metal back reflector to work as the reflective metasurface, and most of these were designed on the basis of the Pancharatnam–Berry (PB) phase principle for the incidences of circularly polarized light [24]. However, there are some limitations in optical system integration for practical applications of the reflective metasurfaces, which can be overcome by transmitted dielectric metasurfaces made of high-index dielectrics, such as silicon (Si) [25,26,27,28] and titanium oxide (TiO_2_) [29,30]. Si has evident absorption in the visible band, and the preparation process of high-cost TiO_2_ also degrades its potential application [31]. Meanwhile, the PB phase approach is inherently limited to circularly polarized light and is unsuitable for linear polarization or for polarization-independent metasurfaces. Thus, it is important to design low-cost, high-efficiency devices for linear polarization incidences in the visible range.

In this paper, we propose a high-efficiency dielectric metasurface based on gallium nitride (GaN) nanobricks (refractive index of 2.4) for manipulating orthogonal linear polarizations simultaneously at the wavelength of 530 nm. GaN was chosen because there is no intrinsic absorption throughout the visible spectrum (GaN’s band gap is about 3.4 eV) [32,33]. The polarization beam splitter (PBS) and metalens were designed successfully by tailoring the geometry of the GaN nanobricks to meet the corresponding required phases. Meanwhile, we also designed a simple polarization analyzer for analyzing the degree of linear polarization (DoLP) of the incidences, which can simultaneously focus the *X*-, *Y*-, 45°, and 135° linear polarizations in spatially symmetric positions. Our results would lead to wide applications in photonic research for visible light.

## 2. Design and Analysis

**Figure 1:**
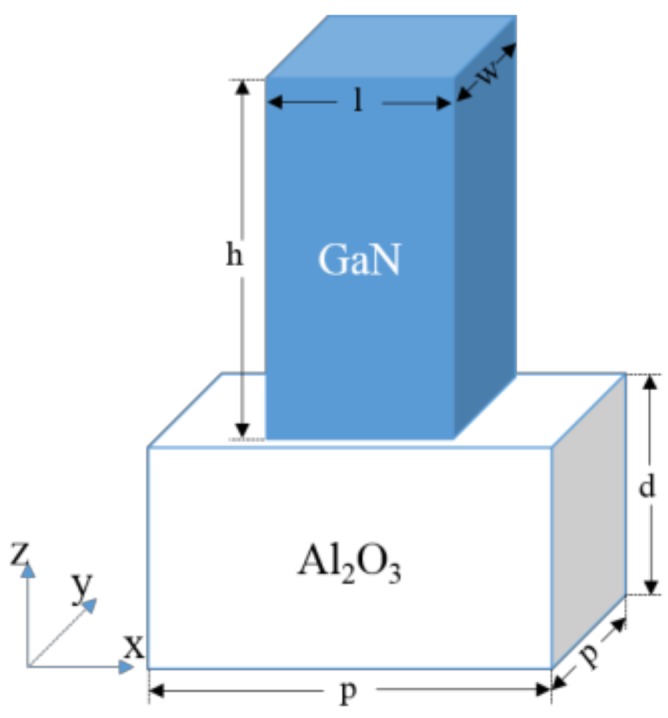
Schematic of the designed unit cell: *p* = 260 nm, *d* = 300 nm, and *h* = 800 nm.

As is schematically shown in Figure 1, the designed GaN nanobrick, with a length, width, and height of *l*, *w*, and *h*, respectively, is adhered onto an Al_2_O_3_ substrate with a depth of *d*. In our designs, the phase accumulation is achieved through the waveguiding effect, which is proportional to the height (*h*) of the designed nanobricks; thus the aspect ratio of the designed nanobricks should be taken into consideration. For incident light with a wavelength of 530 nm, the height of the GaN nanobrick is set as *h* = 800 nm for obtaining the entire 2π modulating phase for the transmitting light, which was optimized and confirmed by the finite-difference time-domain (FDTD) method. For simplifying the calculation, we set the depth of the Al_2_O_3_ substrate as *d* = 300 nm. The concrete modulating phases of the transmitted linear-polarized light could be realized by changing the size of the GaN nanobricks. A square lattice with a lattice constant of *p* = 260 nm was chosen for the optimized and designed simulations; this was obtained from the following two standards: (i) the lattice constant must be less than half of the main working wavelength to avoid the diffraction effect; (ii) the lattice constant should be large enough for the near-field strong interactions between two neighboring nanobricks to be avoided [34]. The length and width of rectangular nanobricks affects the transmission amplitudes and phases of *X*- and *Y*-polarization, respectively.

**Figure 2:**
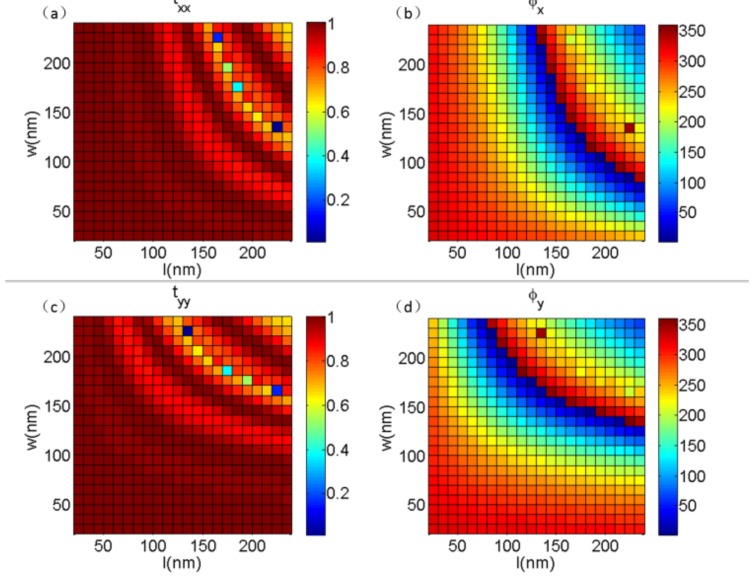
Transmitted light normalization: (**a**) Transmittance variation for gallium nitride (GaN) nanobricks on Al_2_O_3_ substrate, and (**b**) phase as a function of *l* and *w* for normal incidence of the *X*-polarized light. (**c**,**d**) The normalized transmittance and phase as a function of *l* and *w* for normal incidence of the *Y*-polarized light, respectively.

Here, on the basis of double-phase modulation, we introduce the design of the metasurfaces with the GaN nanobricks, which can expand the modulating dimensions of linear-polarized light. In the numerical simulations, periodic boundary conditions were used in the *X*- and *Y*-directions to ensure the accuracy of the phase spectrum in varying structural dimensions. We used the transmission phase of co-polarization light, because of its rectangular structure and the small polarization conversion for the nonrotating periodic nanobricks. As shown in Figure 2a,b, with a normal incident wavelength of 530 nm for *X*-polarized light, the transmittance and the modulating phase of the transmitted *X*-polarized light could be expressed as functions of the length (*l*) and width (*w*) from 30 to 230 nm of the rectangular nanobricks, respectively. It is clear that the modulating phase of the transmitted light could almost cover the entire 2π completely, while the transmittance was close to unity. Similarly, Figure 2c,d represents the transmittance and modulating phases of transmitted *Y*-polarized light, respectively. It can be seen from Figure 2 that for any desired modulating phases of the transmitted *X-* and *Y*-polarized light, there always existed one rectangular GaN nanobrick that could simultaneously satisfy the needs of two orthogonally polarized modulating phases but keep the transmittance close to unity. Therefore, the method used can obtain different phase responses for orthogonal polarization states simultaneously by changing the length and width of the rectangular nanobricks, and the local modulating phases for a couple of incident orthogonal polarization states can be manipulated independently. This is the method with which we can increase the modulating dimension, which can be called the principle of double-phase modulation.

**Figure 3:**
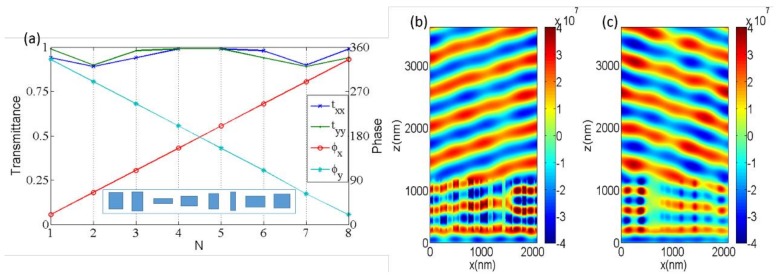
(**a**) The normalized transmittance and the phase of transmitted light through the eight unit cells for the *X*- and *Y*-polarized light incidences at wavelength of 530 nm. The inset in (**a**) is a supercell composed of eight unit cells. (**b**,**c**) The electric field distributions for *X*- and *Y*-polarized light, respectively; it can be clearly seen that *X*- and *Y*-polarized light is refracted into two different directions.

To prove that our proposed method can be applied to optical wavefront shaping, we first designed a polarization beam splitter (PBS) to deflect the *X*- and *Y*-polarized light into different directions efficiently. In order to split the orthogonal polarized light, for simplicity, we designed the device with a couple of opposite gradient phases for *X*- and *Y*-polarized light, which meant *X*- and *Y*-polarized light would be reflected into two opposite directions. As shown in Figure 2, both of the 2π transmitted modulating phases could be obtained by changing the dimensions of *l* and *w* under the *X*- and *Y*-polarized incidences, which meant that any combination of the modulating phases for the *X*- and *Y*-polarized incidences could be realized by a GaN nanobrick with a certain length and width. On the basis of the generalized Snell’s law [35]:

(1) $$n_{t}\sin\theta_{t}-n_{i}\sin\theta_{i}=\frac{\lambda_{0}}{2\pi }\frac{d\varphi }{dx}$$

reasonable choices of parameters can control the angle of refraction, where $\theta_{i}$ and $\theta_{t}$ are the incident angle and refractive angle, respectively; $n_{i}$ and $n_{t}$ are the refractive indices in the incident and refracted region, respectively; $\lambda_{0}$ is the incident wavelength in vacuum; and dφ/dx is the phase gradient. It is worth mentioning here that dφ and dx are the phase difference and distance difference between two adjacent unit cells. We can utilize the last term in Equation (1) to obtain the desired angle of refraction if the wavelength of the incident light is determined. As shown in Figure 3a, eight unit cells were selected to construct a combination of the modulating opposite phases for the *X*- and *Y*-polarized incidences, and the phase differences were π/4 and −π/4 between two adjacent unit cells for covering the entire 2π, which could be determined from Figure 2. Meanwhile, the transmittance of each unit cell was close to unity to ensure that the designed device was as efficient as possible. The inset in Figure 3a shows a supercell composed of eight unit cells (GaN nanobricks). Theoretically, 45° linear-polarized light can be decomposed into *X*- and *Y*-polarized components. Therefore, if the 45° linear-polarized light is incident to the designed beam splitter, the *X*- and *Y*-polarized components will be deflected into two different directions. As shown Figure 3b,c, the concrete simulated electric fields demonstrate that the transmitted *X*- and *Y*-polarized light was refracted into two opposite directions because of the designed opposite-phase modulation difference for the transmitted *X*- and *Y*-polarized light. It should be noted that the reflected field intensity was almost zero, as depicted in Figure 3b,c, which ensured the high-efficiency transmitted characteristics of the designed GaN metasurface.

**Figure 4:**
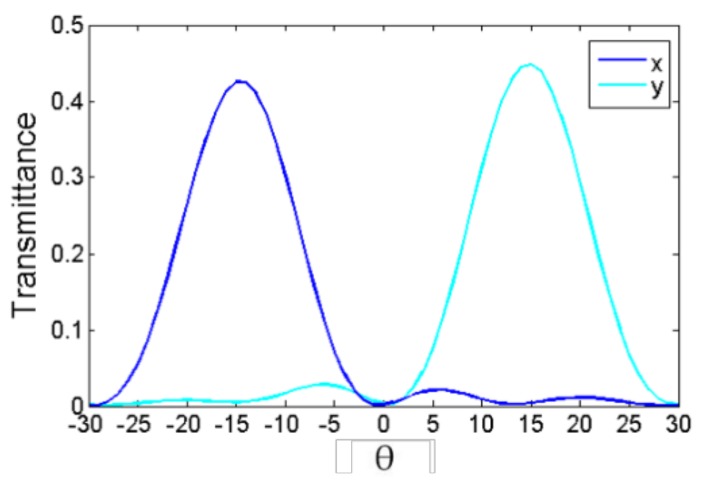
The transmittance of *X*- and *Y*-polarized light as a function of deflection angle (θ) under 45° polarized light incident on the bottom.

After calculating the results from Figure 3b,c, the refraction angle was ±14.7° for the *X*- and *Y*-polarized light, which agreed well with the theoretical value of ±14.76° from Equation (1). The transmitted efficiencies of the *X*- and *Y*-polarized light were 85% and 89.5%, respectively, when the light was incident to the designed device normally, where the deflection efficiency is defined as the ratio of transmitted power to incident power. As shown in Figure 4, when the 45°polarized light was incident to the designed device normally, the deflection efficiencies of the *X*- and *Y*-polarized light were 42.5% and 44.7%, respectively. There were some slight differences in the deflection efficiencies of the *X*- and *Y*-polarized components, which could be attributed to the non-symmetrical characteristics of the nanobrick along the 45° direction.

**Figure 5:**
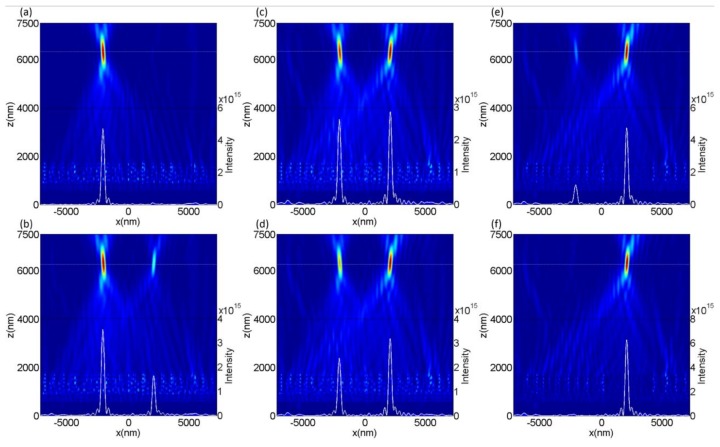
The distribution of transmitted intensities (|*E*|^2^) under the linear polarization states of incident light are (**a**) 0°, (**b**) 30°, (**c**) 42°, (**d**) 45°, (**e**) 60°, and (**f**) 90°. The white solid and dashed lines are the intensity distribution curve and the position of focal plane.

In order to demonstrate the superiority of our designs, on the basis of the principle of double-phase modulation, a difunctional metalens was also designed at the wavelength of 530 nm. Similarly to the PBS, the designed difunctional metalens can focus *X*- and *Y*-polarized light into two different places. The required phase for the focusing lens can be expressed as follows:

(2) $$\varphi (x,y)=\frac{2\pi }{\lambda }(\sqrt{{\left(x\pm x_{0}\right)}^{2}+{\left(y\pm y_{0}\right)}^{2}+f^{2}}-f)$$

where λ is the incident wavelength in vacuum, $x_{0}$ is the horizontal distance between the focus and the center of the metalens, and f is the focal length. Here, we set y=0 , $y_{0}=0$, $x_{0}=8p=2080$ nm, and the f=9λ=4770  nm. We combined $\varphi_{x}$ and $\varphi_{y}$ to select 56 nanobricks for the metalens in Figure 2; the locations of the focuses of the *X*- and *Y*-polarized light were $\left(-x_{0},0,f\right)$ and $\left(x_{0},0,f\right)$. With the normal incidences at the wavelength of 530 nm, Figure 5a–e demonstrates the distributions of the different transmitted intensities of the designed difunctional metalens under 0° (*X*-polarization), 30°, 42°, 45°, 60°, and 90° (*Y*-polarization) linear-polarized states of incident light, respectively. We also extracted the transmitted intensity distributions in the *X–Y* plane at the focal planes under the incidences of different linear polarizations, as shown in the lower half of each figure in Figure 5. As shown in Figure 5a,f, when 0° (*X*-polarization) and 90° (*Y*-polarization) linear-polarized light was incident to the designed metalens, there was just one focusing spot each at the positions of (−2080 nm, 0, 6470 nm) and (2080 nm, 0, 6470 nm), respectively, in the transmitted fields (the ordinates of the focuses (in *Z*-direction) were larger than the setup of 4770 nm because of the height of the metasurface and an additional 600 nm air layer). Because arbitrary linear polarizations can be decomposed into two orthogonal linear polarizations, thus we could approximately analyze the incident linear polarization state on the basis of the focusing intensity. For example, for the 30° linear polarization incidence, compared to the *Y*-polarized component, the *X*-polarized component was dominant; thus the focusing energy of *X*-polarized light was stronger than that of *Y*-polarized light, as shown in Figure 5b. Similarly, for the 60° linear polarization incidence, the focusing intensity of *X*-polarized light was weaker than that of *Y*-polarized light, as depicted in Figure 5e. Meanwhile, for the incidence of 45° linear polarization, as shown in Figure 5d, the focusing intensities of *X*- and *Y*-polarized components in the transmitted fields should be the same, but there was a small mismatch in the two focuses’ energies, which could be attributed to the non-symmetrical characteristics of the nanobrick along the 45° direction and which may have led to a very small intensity deviation for the intensity responses of *X*- and *Y*-polarized light. After calculation, the numerical aperture (NA) of the metalens designed was 0.836 on the basis of $\mathrm{NA}=\sin[tan^{-1}(D/2f)]$, where *D* and *f* are the size and focal length of the lens. The NA can be made larger by increasing the size of the lens when the focal length is fixed, and the focus effect will be better.

**Figure 6:**
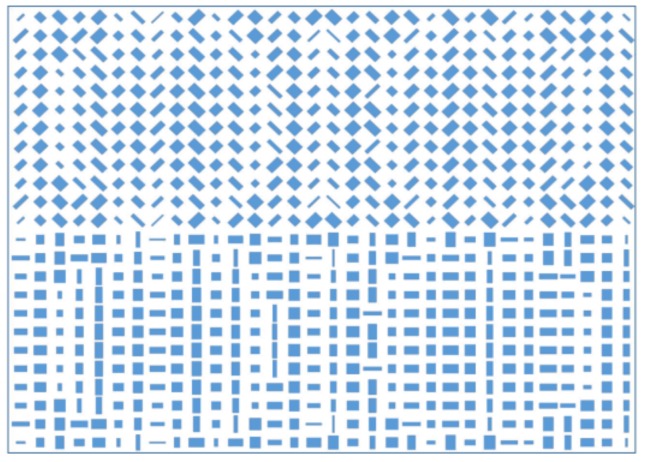
Schematic of the structure array of the designed 3D metalens.

**Figure 7:**
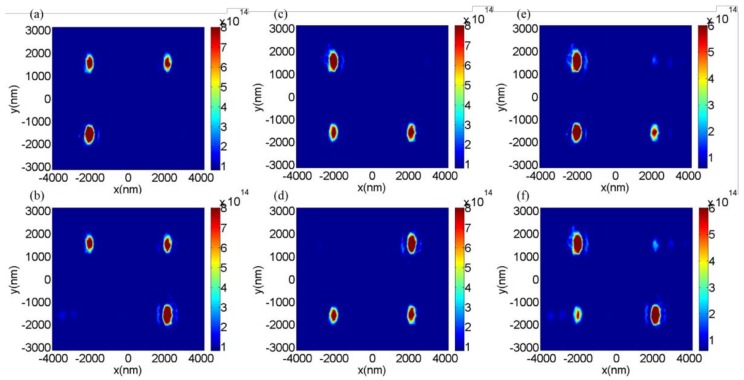
The distributions of transmitted intensities (|*E*|^2^) in the focusing plane (*X–Y*) under (**a**) *X*-, (**b**) *Y*-, (**c**) 45°, (**d**) 135°, (**e**) 30°and (**f**) 60° linear-polarized incidences.

To further confirm the versatility of the design method for analyzing arbitrary linear polarizations, a three-dimensional (3D) metalens was designed for analyzing *X*-, *Y*-, 45°, and 135° linear-polarized light simultaneously. On the basis of Equation (2), here, we set $x_{0}=8p=2080$ nm, $y_{0}=6p=1560$ nm, and f=5λ=2650 nm, and we set the focusing points as $\left(-x_{0},-y_{0}\right)$, $\left(x_{0},-y_{0}\right)$,$\left(-x_{0},y_{0}\right)$, and $\left(x_{0},y_{0}\right)$ for the X-, Y-, 45°, and 135° linear-polarized light, respectively. The GaN nanobrick array of the designed 3D metalens is schematically shown in Figure 6, in which 768 GaN nanobricks were selected for satisfying the phase requirements on the basis of the phase distributions in Figure 2. The size of the designed metalens is 8.32 µm × 6.24 µm; there are two parts of the GaN nanobrick array (the lower part is used to focus *X*- and *Y*-linear-polarized light to the locations of $\left(-x_{0},-y_{0}\right)$ and $\left(x_{0},-y_{0}\right)$, respectively; the upper part is obtained by rotating the GaN nanobricks in the lower part to 45° and −45° accordingly for manipulating and focusing the 45° and 135° linear-polarized light to the locations of $\left(-x_{0},y_{0}\right)$ and $\left(x_{0},y_{0}\right)$ respectively. The transmission intensity distributions in the focal plane are demonstrated in Figure 7a–f under the incidences of the *X*-, *Y*-, 45°, 135°, 30°, and 60° linear-polarized light, respectively. We can observe that the focuses were oval, which could be attributed firstly to the horizontal and vertical axes of the scale not being the same for the focus, as well as that the longitudinal structural elements provided a smaller phase distribution than that in the lateral direction. As shown in Figure 7a, under the incidence of *X*-polarized light, the intensity at the position of $\left(x_{0},-y_{0}\right)$ for the *Y*-polarization focus was nearly zero, as there was no *Y*-polarized component in the incidence, but the focusing intensities for the 45° and 135° linear-polarized components were nonzero as a result of the decomposed components from the *X*-polarized incidence. This was similar for arbitrary linear-polarized incidences. For example, in Figure 7f, under the incidence of 60° linear-polarized light, there were four evident focusing points because of the nonzero decompositions of the *X*-, *Y*-, 45°, and 135° linear-polarized components.

**Figure 8:**
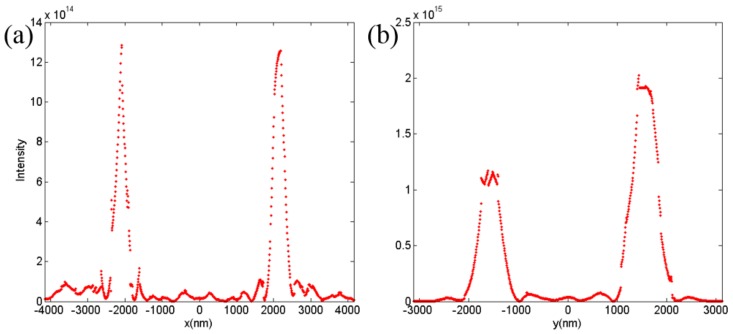
The transmitted intensity (|*E*|^2^) profiles in focal plane at (**a**) *y* = −1560 nm and (**b**) *x* = −2080 nm under the incidence of 45° linear-polarized light.

Figure 8a,b illustrates the normalized transmitted intensity distribution in the focal plane (y=−6p, x=−8p) in case of the 45° linear-polarized light incidence. As shown in Figure 8a, the intensity was almost undifferentiated, which agreed well with Figure 7c. The *X*-polarized focusing intensity was half that of the 45° linear-polarized light, as depicted in Figure 8b, because the 45° linear-polarized light could be decomposed into *X*- and *Y*-polarized components. The difference in the full width at half maximum between the *X*-polarized focus and 45° linear-polarized focus proves that the vertical phase profile was smaller once again, as mentioned before, which could be solved by increasing the size of the lens. The designed 3D metalens can be used as a polarization splitter to separate arbitrary linear polarizations into two pairs of orthogonal polarizations, and it can also be used as a linear polarization generator under the incidence of any type of polarized light.

In addition, by measuring the focusing energies at each focal point of the designed 3D metalens, we could analyze the polarization states of the arbitrary linear-polarized light. The focusing energies of *X*-, *Y*-, 45°, and 135° linear-polarized components can be expressed as *I_x_*, *I_y_*, *I*_45_, and *I*_135_, respectively, from which we could analyze the polarization direction of arbitrary linear-polarized incidences. When the incident linear polarization angle is θ, the focusing energies of the four focal points are $I_{x}^{\prime }$, $I_{y}^{\prime }$, $I_{45}^{\prime }$, and $I_{135}^{\prime }$, respectively. The Stokes parameters of *I*, *Q*, and *U* and the DoLP of the incidence can be obtained as follows:

$$s_{1}=\frac{I_{x}^{\prime }-aI_{y}^{\prime }}{I_{x}^{\prime }+aI_{y}^{\prime }},\;s_{2}=\frac{I_{45}^{\prime }-bI_{135}^{\prime }}{I_{45}^{\prime }+bI_{135}^{\prime }},\;DOLP=\frac{\sqrt{s_{1}^{2}+s_{2}^{2}}}{I_{total}}$$

where $a=I_{x}/I_{y}$, $b=I_{45}/I_{135}$, and $I_{total}$ is the total intensity of transmitted light. The Stokes parameters can be normalized to make the unit total light intensity:

$$Q=\frac{s_{1}}{\sqrt{s_{1}^{2}+s_{2}^{2}}},\;U=\frac{s_{2}}{\sqrt{s_{1}^{2}+s_{2}^{2}}},\;I=\sqrt{Q^{2}+U^{2}},$$

and the polarization direction can be expressed as $\tan2\theta =\frac{U}{Q}$.

Here, we calculated the power value instead of energy for analyzing the polarization direction of the incidences under simulation. When light with a polarization angle of *θ* was incident to the designed polarization analyzer, the powers of the *X*-, *Y*-, 45°, and 135° polarized components could be obtained at their focuses accordingly, and then we could calculate the polarization angle of the incident light. For example, when light with polarization angles of *θ* = 30° and *θ* = 60° was incident to the designed polarization analyzer, the powers of the *X*-, *Y*-, 45°, and 135° polarized components could be obtained as is depicted in Table 1. We calculated the polarization angles of the incident light as 29.87° and 59.53°, respectively, which agreed well with the true values. This means that the designed 3D metalens can be used for analyzing linear polarization states with high accuracy. If arbitrary linear-polarized light is incident, we can calculate the Stokes parameters *I*, *Q*, and *U* by collecting the energy at each focal point and then analyze the polarization direction of the incident linear-polarized light.

## 3. Conclusions

In summary, we propose a metasurface based on the novel dielectric material GaN to realize high-efficiency modulation for both of the orthogonal linear polarizations simultaneously in the visible range. We designed a beam splitter to refract the light of orthogonal polarizations into two opposite directions, as well as a metalens to focus the *X*- and *Y*-polarized light in two spatially symmetric positions. We also designed a 3D metalens that can simultaneously focus *X*-, *Y*-, 45°, and 135° linear polarizations in spatially symmetric positions, which can be applied as a simple polarization analyzer for analyzing the polarization direction of the incidences. Our presented method can be used over the whole visible range, which gives it great potential in applications such as polarization imaging. It is promising that the designed devices can be integrated into systems to form a highly integrated polarization multiplexor.

## Figures and Tables

**Table 1:** The power values of *X*-, *Y*-, 45°, and 135° linear-polarized components at focus.

*θ*	*P_x_*	*P_y_*	*P*_45_	*P*_135_
30°	0.213	0.089	0.300	0.032
60°	0.079	0.245	0.295	0.034
